# Supplementary material for: Computational geometry analysis of dendritic spines by structured illumination microscopy
Source: Nat Commun. 2019 Mar 20;10:1285. doi: 10.1038/s41467-019-09337-0 (PMC6427002; doi:10.1038/s41467-019-09337-0)
Supplement: Supplementary file 1 — Supplementary Information [file 41467_2019_9337_MOESM1_ESM.pdf]

Supplementary Information

Computational geometry analysis of dendritic spines by structured illumination microscopy

Kashiwagi et al.

Supplementary Table 1

	SVC	Operator A	Operator B	Operator C	Operator D (training dataset)
SVC		80%	78%	79%	92%
Operator A			79%	80%	86%
Operator B				80%	78%
Operator C					82%

The results of spine classification into mushroom and non-mushroom spines by four operators (A to D; all have sufficient experience in spine shape evaluation). The classification result of the operator D was used for training SVC. The percentages of matched spines over total spines (n = 400) were calculated.

Supplementary Table 2

Figure	Source	df	F	*p*	Spines	Cells	Cultures
4a	Neighbor	5, 120	1.155	0.3356	14	4	4
4a	Stimulated	5, 120	5.865	6.96×10^{-5}	14	4	4
4d	Neighbor	6, 93	1.535	0.1754	10	4	4
4d	Stimulated	6, 53	6.073	6.87×10^{-5}	6	4	4
4f	control-Fc	8, 63	5.997	1.03×10^{-5}	6	4	4
4f	Nrx1β-Fc	8, 63	0.7677	0.6322	6	5	3
4g	control-Fc	10, 41	4.774	1.532×10^{-4}	4	3	2
4g	Nrx1β-Fc	10, 54	0.3579	0.9592	5	4	2

Supplementary information of one-way ANOVA followed by Tukey–Kramer procedures for multiple comparison tests presented as the mean ± SEM in Figure 4. Significant at $^{*}p < 0.05$, $^{**}p < 0.01$, $^{***}p < 0.001$.

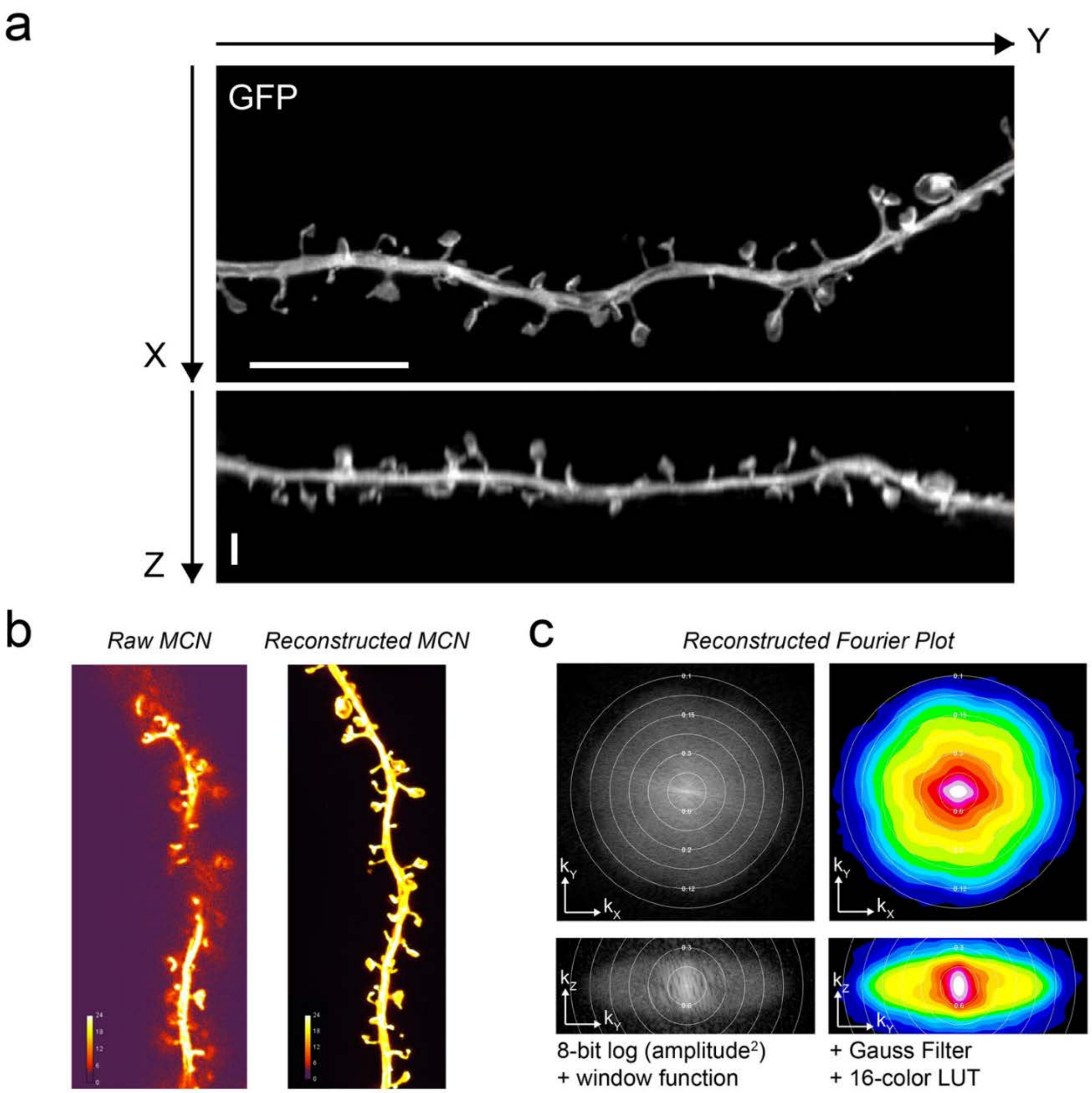

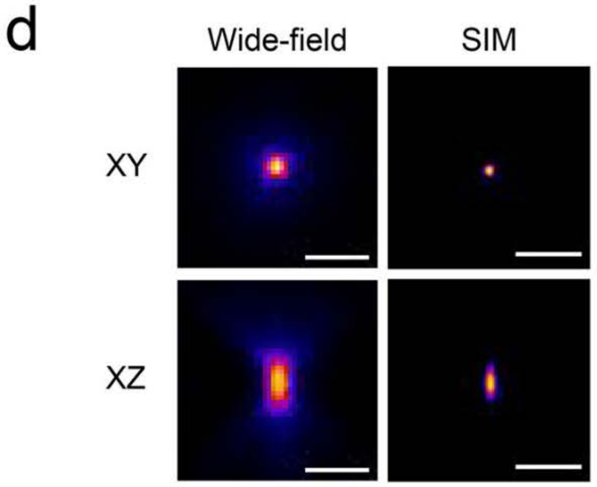

FWHM (nm)	XY	Z
Wide-field	263 ± 7	571 ± 24
SIM	108 ± 4	323 ± 17

Supplementary Figure 1

Evaluation of 3D-SIM image data quality

(a) A representative 3D-SIM image of a fixed hippocampal neuron expressing GFP. A maximum intensity projection image from a reconstructed image stack for a volume 7.56 μm thick. Bars: 5 μm in x-y and 1 μm in y.

(b) Checking and verifying the quality of 3D-SIM data using the SIMcheck plugin for ImageJ. The ratio of the modulation contrast-to-noise (MCN) was calculated from raw 3D-SIM images. The data were translated to a heatmap (raw MCN) by applying a custom color look-up table. The MCN value was combined with the reconstructed SIM image to create a modulation contrast map (reconstructed MCN).

(c) Fourier transform of the reconstructed image using the SIMcheck plugin. Lateral and orthogonal Fourier power spectrum from the unclipped reconstructed data of panel (a), with a window function and no cut-off. The 8 bit log-scaled ($amplitude^2$) Fourier power spectra were blurred, and are shown with a 16-color look-up table. Concentric rings indicate the respective spatial resolution in μm.

(d) Measurement of FWHM of yellow/green fluorescent microspheres (100 nm in diameter) with N-SIM operating in wide-field mode or 3D-SIM mode. Bars: 1 μm in both x-y and x-z. Data are from 3 and 5 beads for wide-field and 3D-SIM respectively. Numbers in the table are mean ± SD.

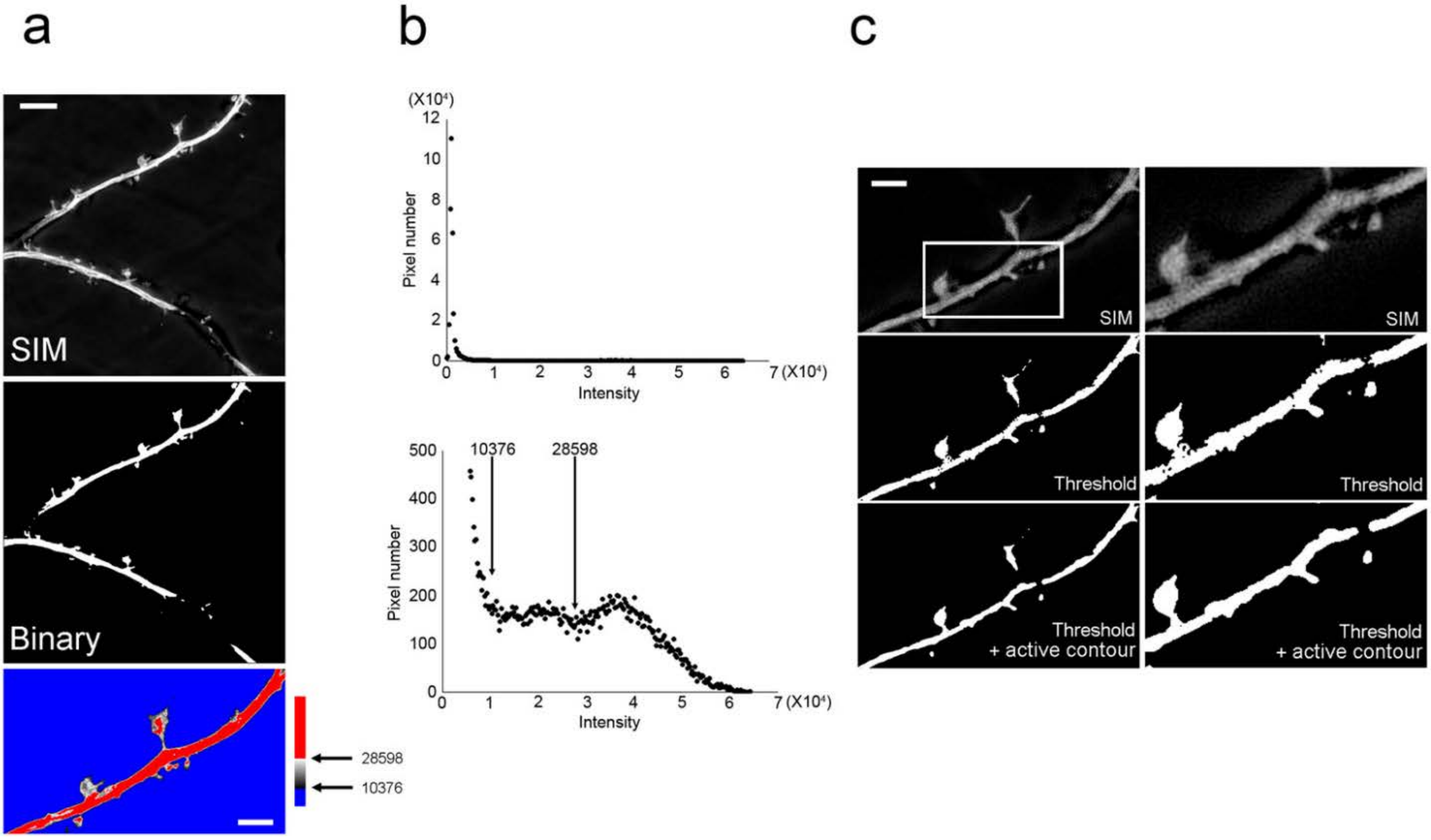

Supplementary Figure 2

Image segmentation by multi-level thresholding and geodesic active contours

(a) Multi-level thresholding of the original SIM image. In this case, the two threshold values were 10376 and 28598. Pixels within the range 10376–28598 are enriched in dendritic spines (pseudocolor image). Bars: 4 μm for low-magnification images, 2 μm for the higher-magnification image.

(b) Plot of pixel frequencies against pixel intensity. The lower graph shows the enlarged profile in the range 0–500 pixels, indicating the presence of a tail above the value of 10378, which corresponds to the pixels within dendritic spines and shafts.

(c) Image manipulation by geodesic active contours. Panels in the right column are enlarged images of the area indicated by a white rectangle. After image manipulation with geodesic active contours, small holes within the dendrite and small islands of pixels outside of the dendrite were effectively eliminated. Bar: 2 μm for low-magnification images, 1 μm for higher-magnification images.

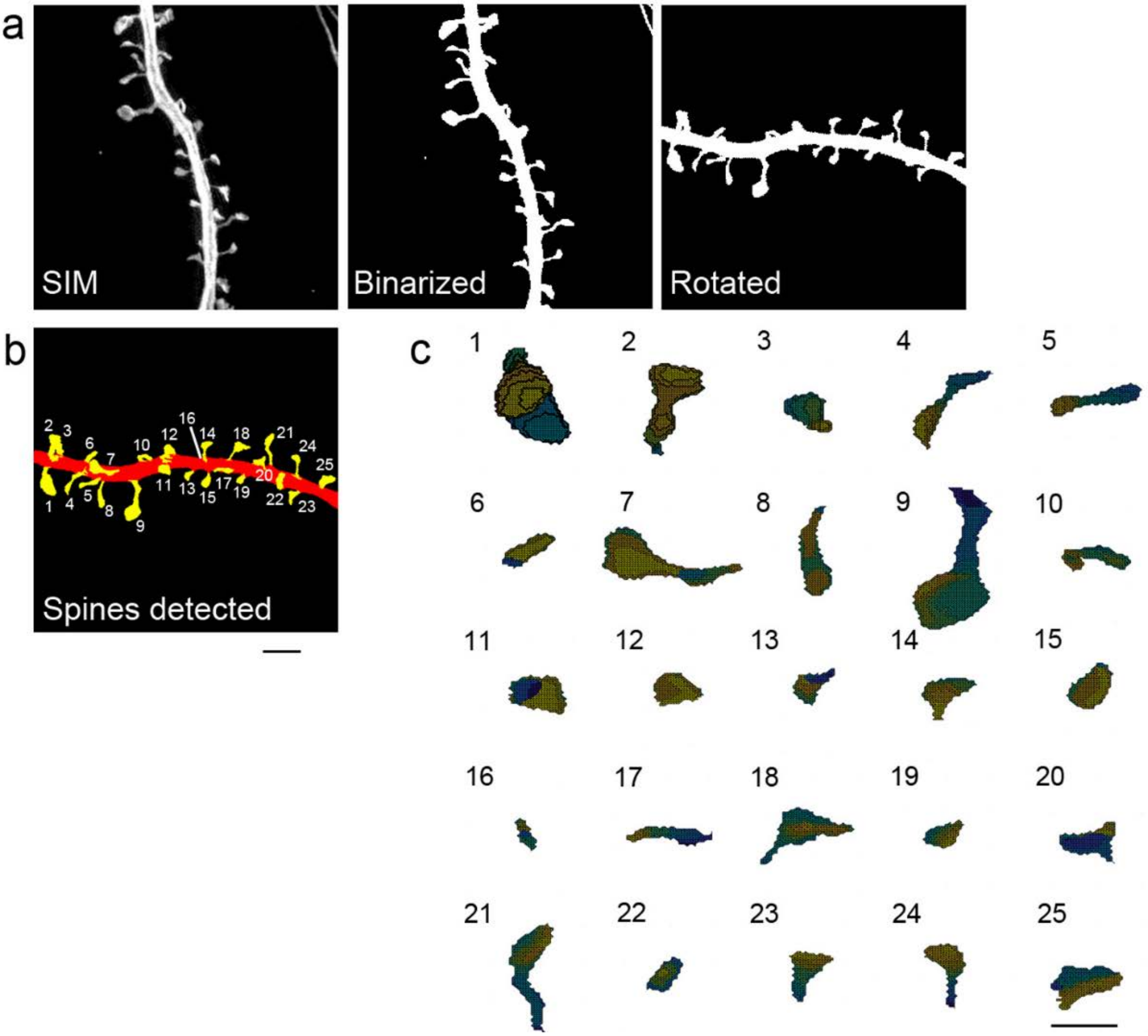

Supplementary Figure 3

Automated detection of spines and generation of polygonal meshes of isosurfaces by marching cube algorithm

(a) Processing steps for SIM images. The raw image stack (SIM) was binarized (Binarized), and the main dendrite was isolated and rotated to be aligned with the horizontal axis in the XY image plane (Rotated).

(b) Pseudocolor image of the same dendritic segment after automatic detection of spines. Yellow pixels correspond to identified spine areas with numbers from 1 to 25. Spines 2 and 3 and spines 14 and 16 overlap in the XY projection image. Bar: 2 μm.

(c) Polygonal meshes of 25 spines identified in panel (b). Pseudocolor indicates z-positions of individual triangular faces (yellow is away from and green is close to the culture substrate). The details of the method of spine identification and the determination of the spine-shaft border for the same dendritic segment were shown in the Supplementary Fig. 4. Bar: 1 μm.

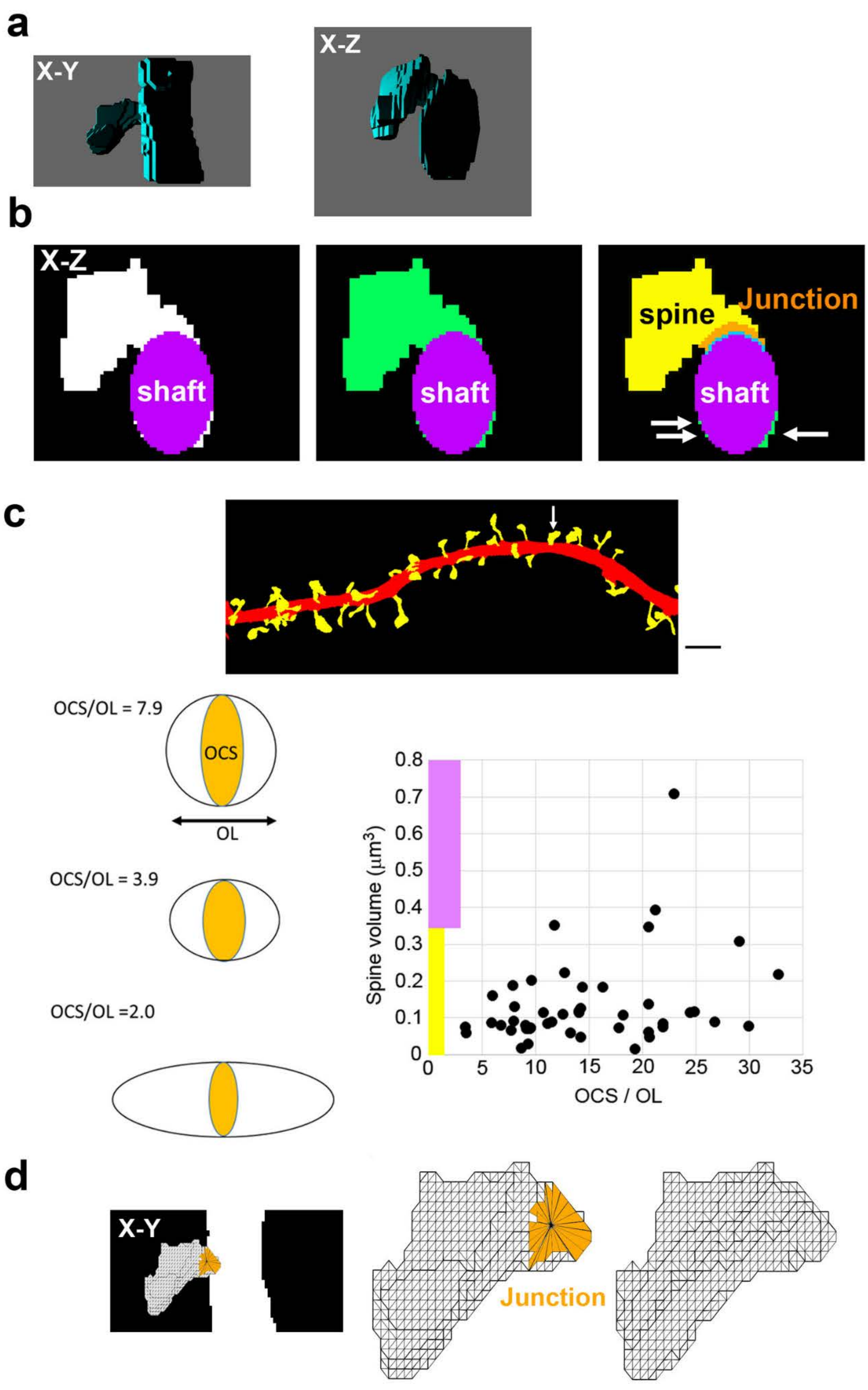

a
X-Y
X-Z
b
X-Z
shaft
shaft
spine
Junction
shaft
c
OCS/OL = 7.9
OCS
OL
OCS/OL = 3.9
OCS/OL =2.0
Spine volume (μm³)
OCS / OL
d
X-Y
Junction

Supplementary Figure 4

Fitting of a dendritic shaft by an elliptic cylinder, determination of a spine-shaft junction and selection of spine candidates by their shapes

(a) A dendritic segment in x-y and x-z views.

(b) Fitting of the dendritic shaft in (a) by an elliptic cylinder. The voxel clusters outside of the fitted cylinder were labeled as spine candidates. Small green voxel clusters marked by arrows were judged to be non-spine objects based on their sizes (< 0.01 μm^3). The spine candidate (corresponds to spine No. 25 in Supplementary Fig. 3) was dilated for two voxels (~60 nm) and the junction between the spine candidate and the dendritic shaft was identified (blue pixels in the right image) as the shaft-side surface. Next the spine candidate voxels within 150 nm from the shaft-side surface were identified (orange pixels in the right image) as the spine-side surface. We finally eliminated the voxels of the spine-side surface. This procedure is effective in removing the voxels that stretch on the surface of shafts away from the spine base.

(c) Upper image shows the spine candidates detected by the above procedure [the arrow indicates the spine analyzed in (a) and (b)]. Among these objects, those with elongated shapes were rejected. Lower left illustration shows examples of objects with different OCS/OL values (OCS: largest cross section, OL: length along longitudinal axis). OCS/OL value is the ratio between object cross section area and its length. Objects with OCS/OL < 2.0 and their volume < 0.32 μm^3 and objects with OCS/OL < 4.0 and their volume > 0.32 μm^3 were rejected. The lower right graph shows the distribution of spine candidates (yellow spine candidates in the upper image) in the two-dimensional space defined by their OCS/OL values and their volumes. The yellow and magenta areas correspond to the rejection areas. All spine candidates are distributed outside of the rejection areas and are judged to be true spines. Bar, 2 μm.

(d) The surface of spine-shaft junction after polygon mesh generation. The orange triangles show the surface of the junction.

a

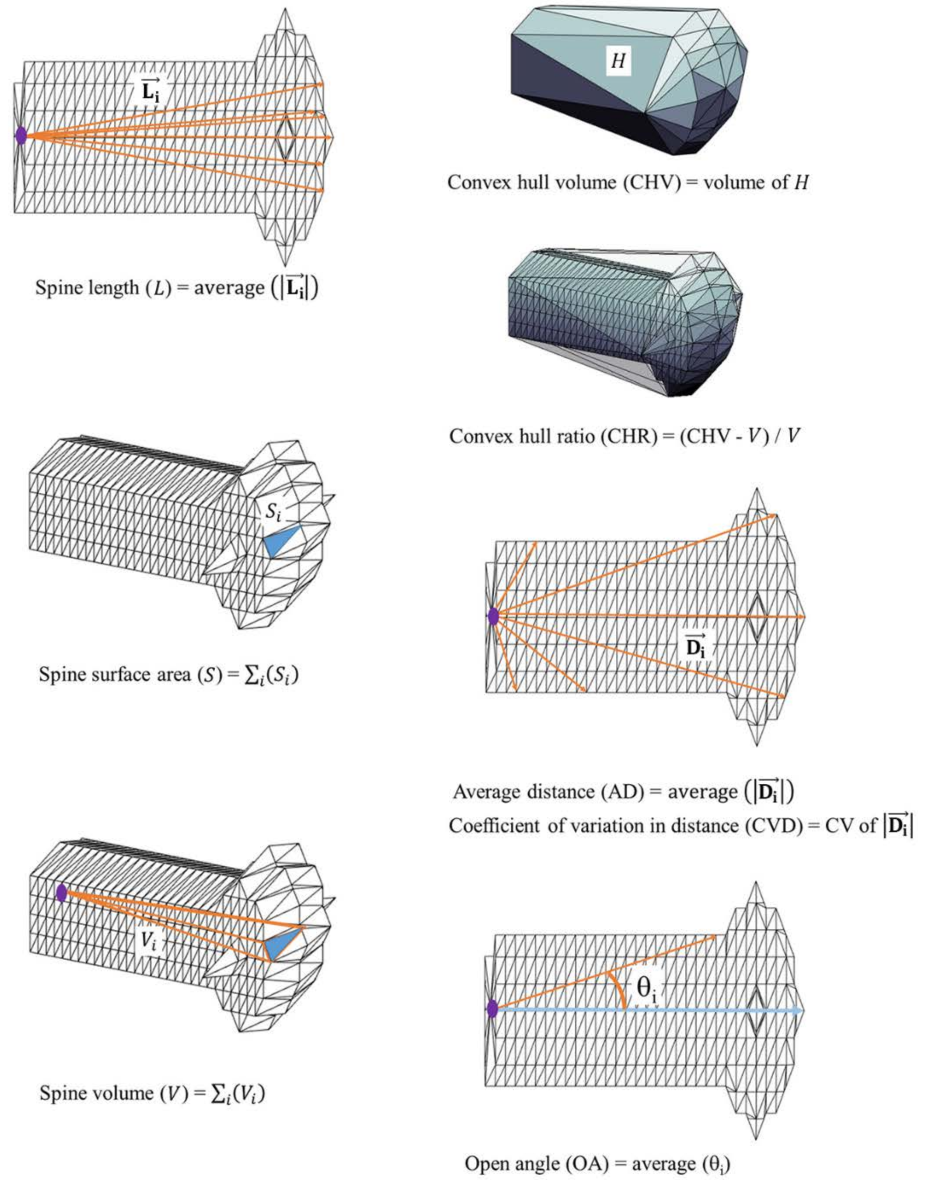

b

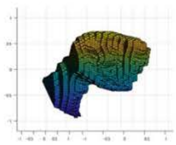

Spine surface area (S) = 7.85

Spine volume (V) = 0.89

Convex hull volume (CHV) = 1.61

Convex hull ratio (CHR) = 0.81

Spine length (L) = 1.96

Average distance (AD) = 1.06

Coefficient of variation in distance (CVD) = 0.45

Open angle (OA) = 0.54

c

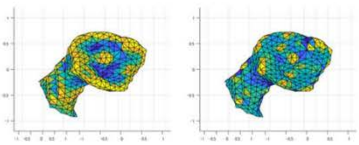

Spine Curvature Data Spine No = 1

Average of Mean Curvature = 2.2580

Variance of Mean Curvature = 58.4250

Average of Gauss Curvature = 2.8978

Variance of Gauss Curvature = 815.4762

Average of Lower 20 percent Mean Curvature = -8.9586

Average of Higher 20 percent Mean Curvature = 12.4059

Average of Lower 20 percent Gauss Curvature = -23.0251

Average of Higher 20 percent Gauss Curvature = 37.2789

Supplementary Figure 5

Schematic explanation of spine geometrical analysis

(a) In this figure, the methods of measuring eight geometrical parameters were described. Spine length (*L*) is average length of vectors with their length larger than the upper 95% of the total vectors from the center of the spine-shaft junction to all surface vertices. Spine surface area (*S*) is summation of the individual triangular surface areas. Spine volume (*V*) is summation of all pyramids with individual triangular surface as their bases and with their apexes located at the center of the spine-shaft junction. Volumes are treated as signed values depending on whether the pyramids are inside or outside of the spine and their summation can be used for calculation of the total volume. Convex hull volume (CHV) is volume of the smallest convex set of vertices that contains the entire spine polygon meshes. Convex hull ratio (CHR) is the relative ratio of the volume inside the convex hull but outside the spine against the spine volume (*V*). Average distance (AD) is the average lengths of all vectors that run from the center of the spine-shaft junction to all the vertices. Coefficient of variation in distance (CVD) is calculated from the population of vectors used in AD calculation. Open angel (OA) is the average angle between individual vertex vectors and their averaged vector which reflects the overall direction of the spine longitudinal axis.

(b) An example of polygonal mesh calculated by Geometric_calculation_program.

(c) An example of polygonal meshes calculated by Geometric_curvature_program.

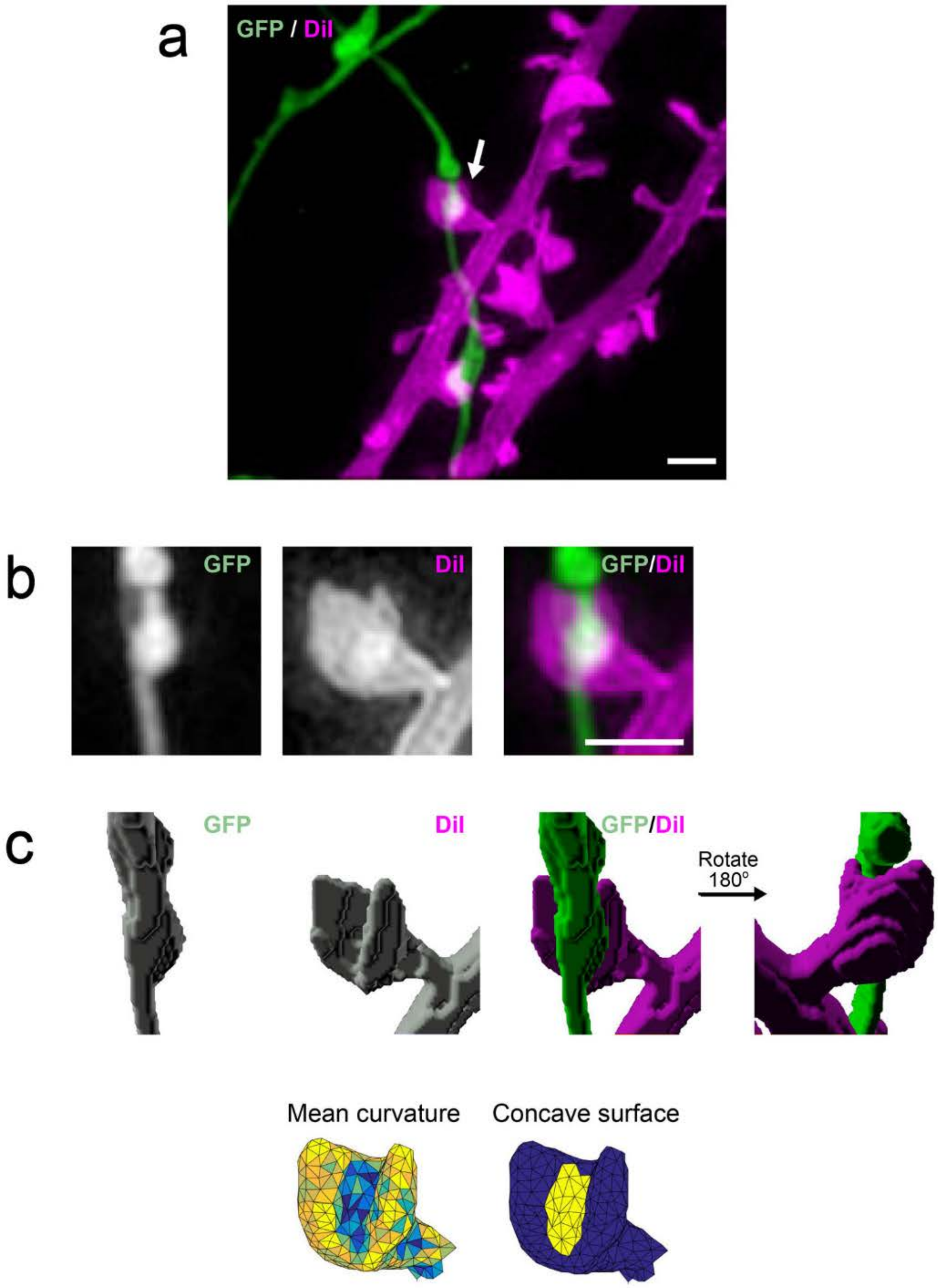

Supplementary Figure 6

Dual-color SIM imaging of dendritic spines and presynaptic axons

(a) Dual-color 3D-SIM imaging of dendrites labeled with DiI (magenta) and axons labeled by GFP (green). GFP-expressing hippocampal neurons were fixed, and nearby putative postsynaptic neurons were loaded with DiI. Contact sites between GFP-positive presynaptic axons and DiI-positive postsynaptic dendrites were identified and imaged. Bar: 1 μm.

(b) Higher-magnification images of a DiI-labeled spine, a GFP-labeled axonal varicosity, and their merged images, marked by an arrow in panel (a). Bar: 1 μm.

(c) Polygonal meshes of the dendritic spine and the axonal varicosity shown in panel (b) (images in the upper row). Surface mean curvature (pseudocolor mapping of the lower left image) and surface area with the smallest negative mean curvature (a yellow area in the lower right image) are also shown.

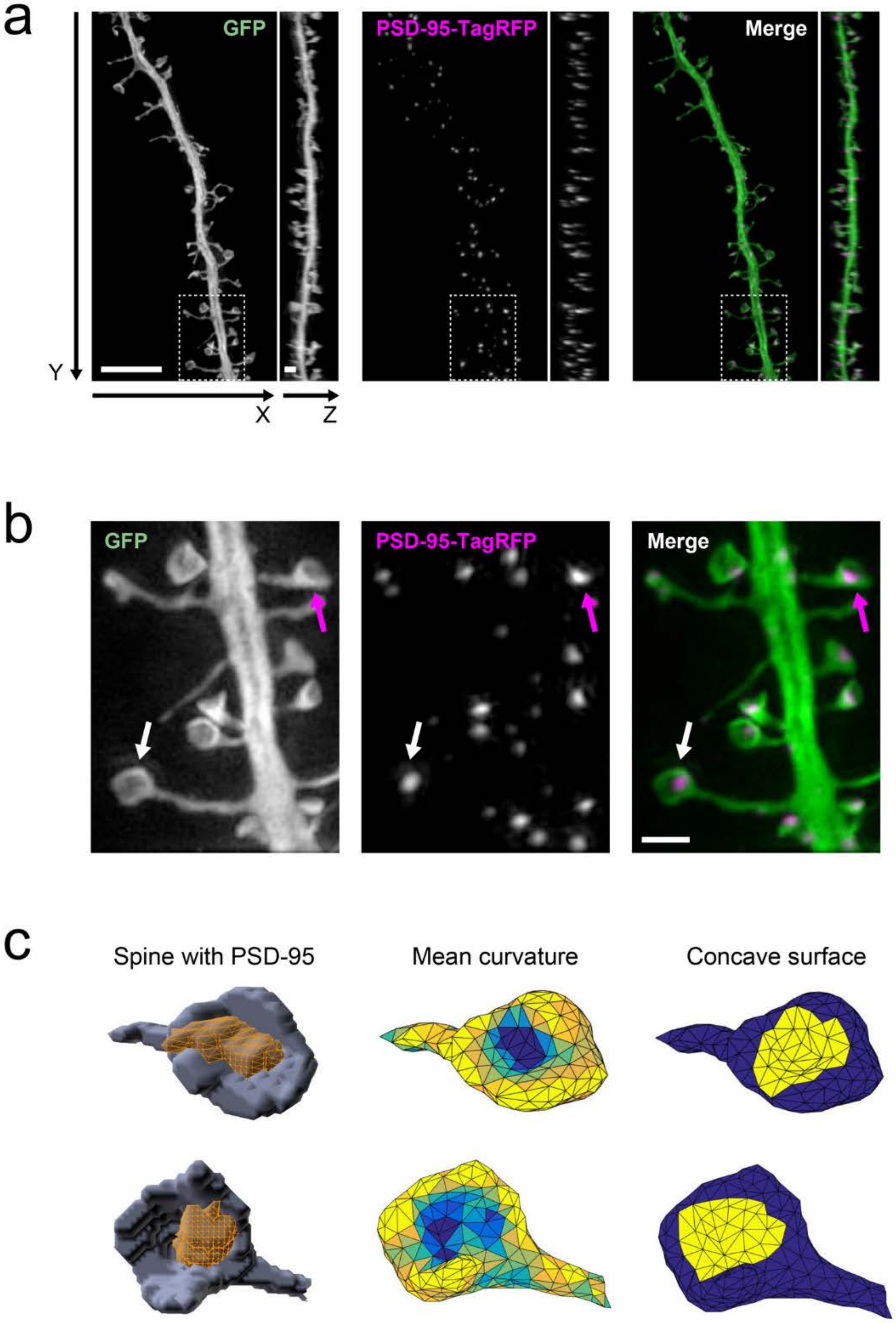
a
GFP
PSD-95-TagRFP
Merge
Y
X
Z
b
GFP
PSD-95-TagRFP
Merge
c
Spine with PSD-95
Mean curvature
Concave surface

Supplementary Figure 7

Dual-color SIM imaging of dendritic spines and postsynaptic density labeled by PSD-95-TagRFP

(a) Dual-color 3D-SIM images of a GFP-filled dendrite (GFP) and PSD-95 tagged with TagRFP (PSD-95-TagRFP). Maximum intensity projection images from reconstructed image stacks for the volume thickness of 5.4 μm. Bars: 5 μm in x-y and 1 μm in y-z.

(b) Higher-magnification images of the dendrite and PSD-95 clusters shown in dashed squares in panel (a). Two typical mushroom-shaped spines with PSD-95-TagRFP are marked by arrows. Bar: 1 μm.

(c) Polygonal meshes of the two dendritic spines marked by arrows in panel (b). Surface mean curvature (pseudocolor mapping in the middle images), surface area with the smallest negative mean curvature (yellow areas in the right images), and PSD-95 clusters mapped onto spine surfaces (brown meshes in the left images). The positions of the concave surfaces matched the PSD-95-TagRFP–positive areas.

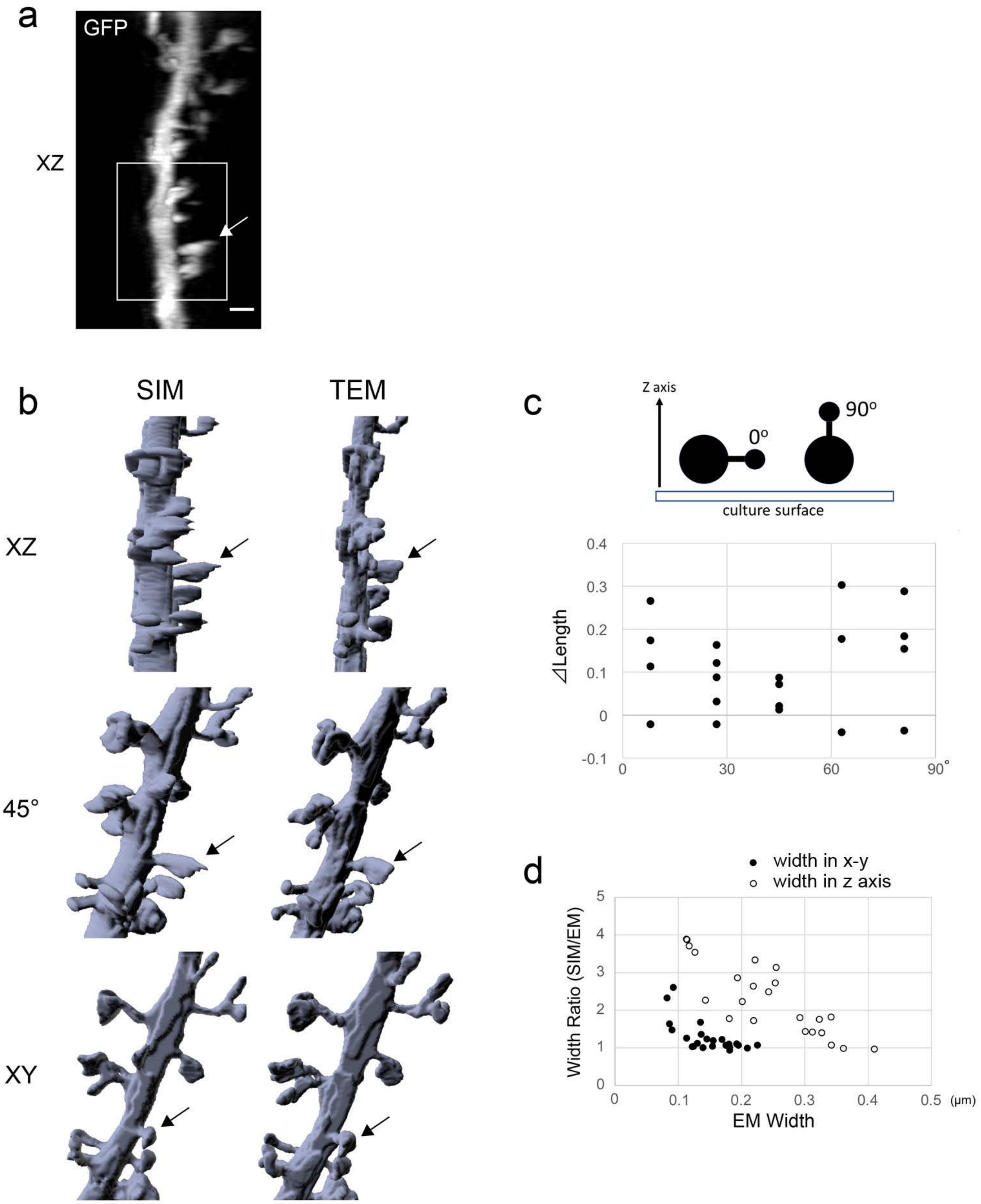
a
GFP
XZ
b
SIM
TEM
XZ
45°
XY
c
Z axis
0°
90°
culture surface
⊿Length
0.4
0.3
0.2
0.1
0
-0.1
0
30
60
90°
d
width in x-y
width in z axis
Width Ratio (SIM/EM)
5
4
3
2
1
0
0
0.1
0.2
0.3
0.4
0.5
(μm)
EM Width

Supplementary Figure 8

Comparison of 3D-SIM images with EM spine reconstruction

(a) A dendritic segment that has spines protruding vertically. This sample was processed for correlative light and electron microscopic analysis. Bar: 1 μm

(b) Surface views of SIM and EM data from different angles. The image area corresponds to the white box in panel (a). Top column shows x-z views of a dendritic segment reconstructed from SIM and EM data. Note that spine shapes (arrows) are preserved in the x-z projection images. Middle column shows the same dendritic segment rotated 45 degrees. Bottom column shows x-y view of the identical dendritic segment. Arrows indicate the spine also marked in panel (a).

(c) Spine lengths were measured from both SIM and EM data and the subtracted values (Δ length) were taken as measurement errors of SIM. These values were plotted after they were grouped by the angles (every 18 degrees) formed between the long axes of spines and the x-y plane (n = 20 spines).

(d) Analysis of spine neck widths in x-y plane and z axis. Spine neck widths were measured for both SIM images and EM reconstruction (n = 21 spines), their ratios (SIM/EM) were calculated and plotted against the widths determined from EM reconstruction. Due to lower resolution of SIM in the axial direction, spine necks with their widths less than 350 nm could not be measured accurately. In x-y plane, the error was smaller and reliable measurements were possible with narrower spine necks (< 150 nm).

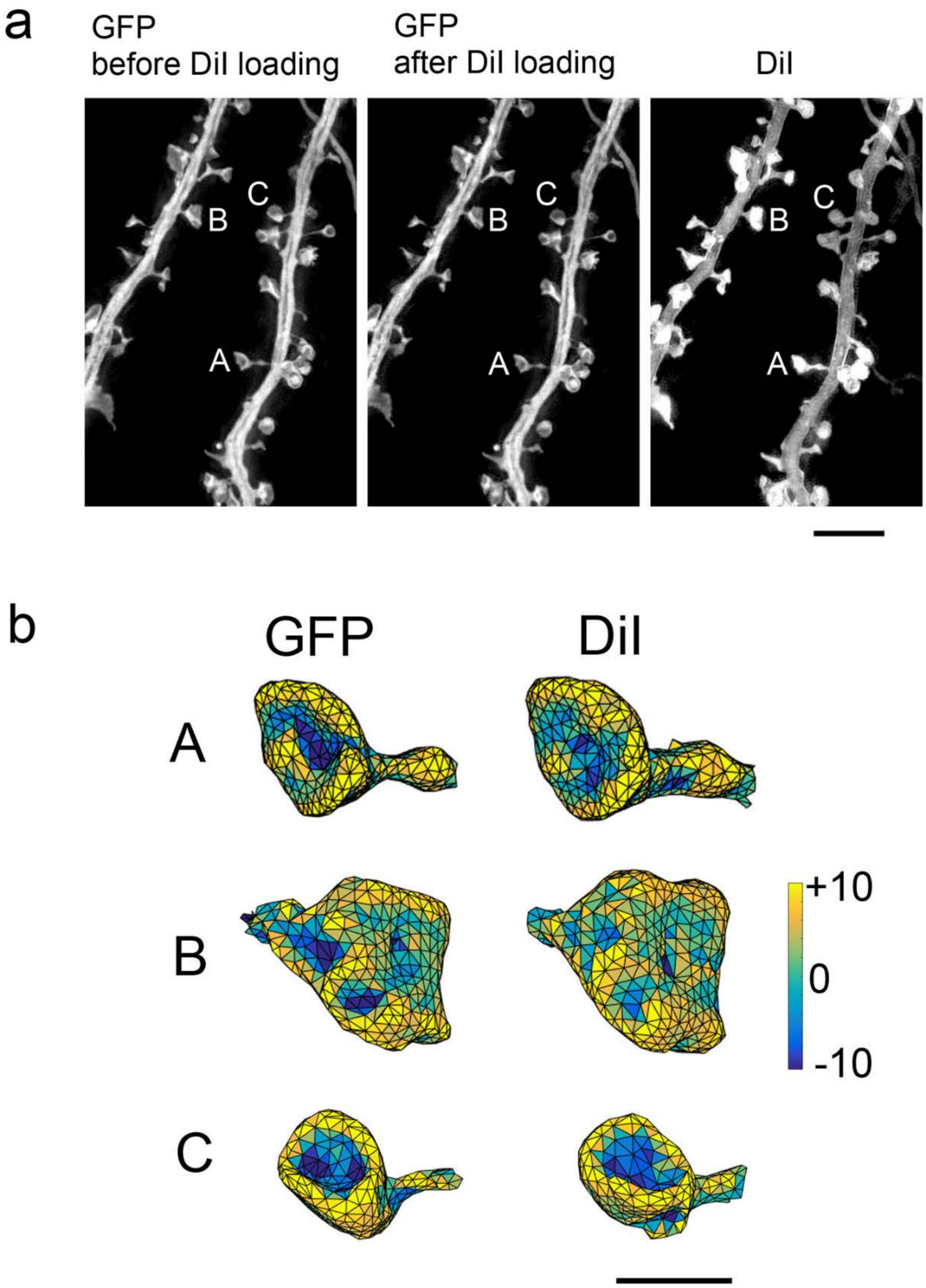

Supplementary Figure 9

Direct comparison of spine morphology by GFP and DiI

(a) SIM images of dendritic segments expressing GFP before and after DiI loading, together with a SIM image generated from DiI fluorescence. Application of DiI did not change spine morphology. Relative spine fluorescence against dendritic shafts in DiI images is higher than that in GFP images, and this difference is likely to be derived from the higher surface-to-volume ratio in spines. Bar: 4 μm.

(b) Reconstructed spine morphology from GFP or DiI fluorescence. Polygonal meshes of three spines (A, B, and C) in panel (a) are shown. Bar: 1 μm.

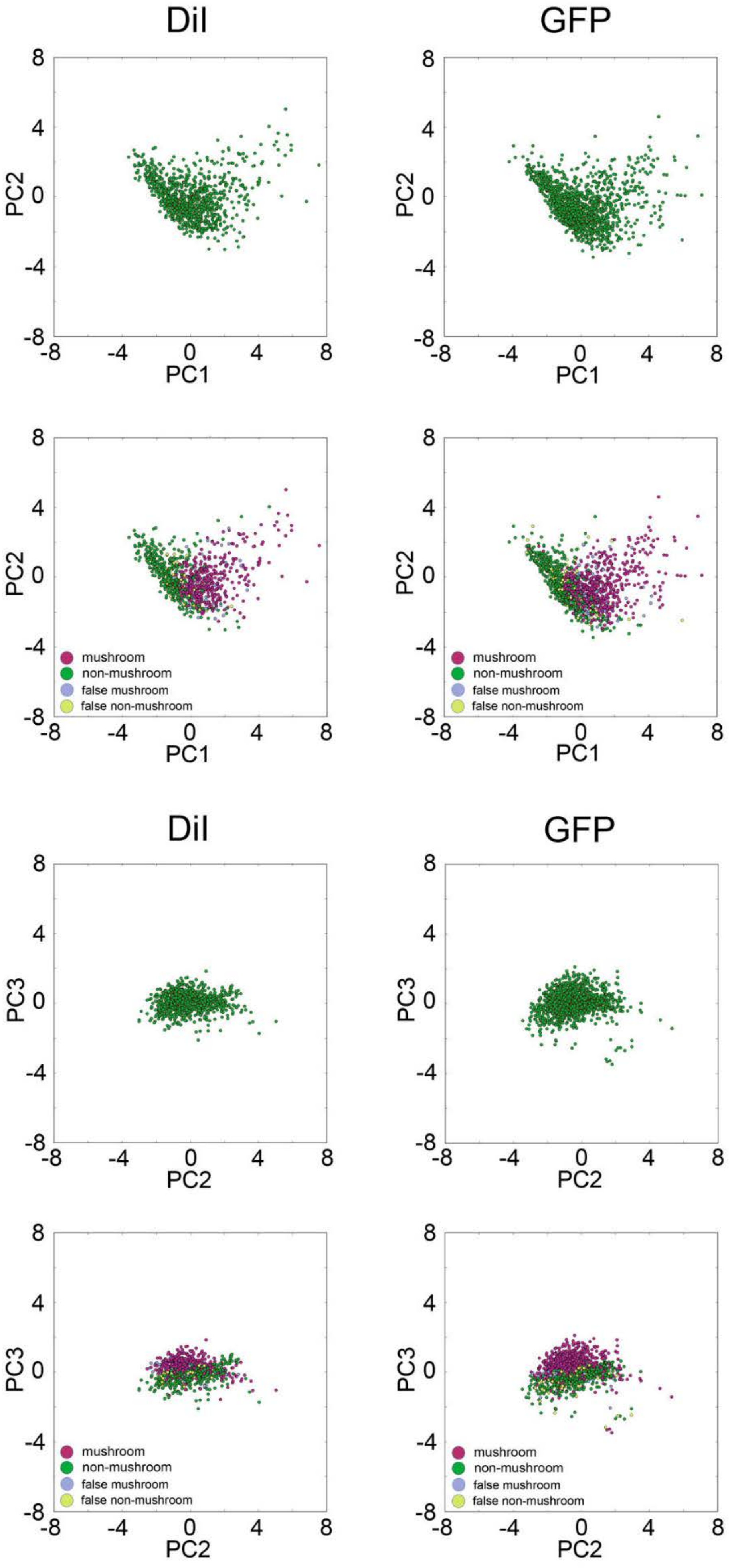
DiI
GFP
PC1
PC2
PC3
mushroom
non-mushroom
false mushroom
false non-mushroom
DiI
GFP

Supplementary Figure 10

Comparison of PCA transformation and SVM-based classification of spine population data obtained from GFP-expressing and DiI-loaded neurons

Distribution of individual spines within the feature space was comparable between GFP-expressing neurons and DiI-loaded neurons. DiI data are from 914 spines, and GFP data are from 1335 spines.

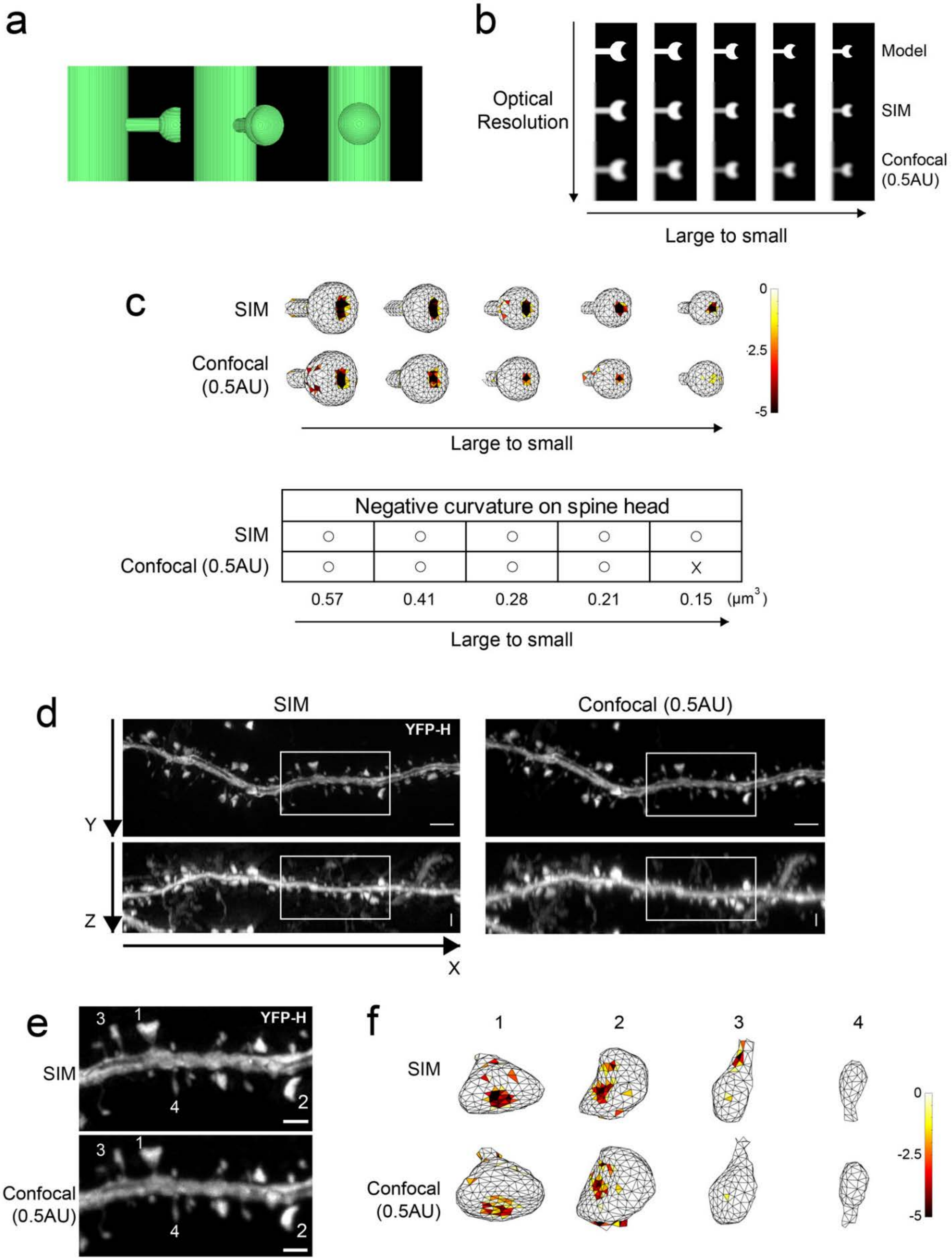

a
b
Optical Resolution
Model
SIM
Confocal (0.5AU)
Large to small
c
SIM
Confocal (0.5AU)
0
-2.5
-5
Large to small
Negative curvature on spine head
SIM ○ ○ ○ ○ ○
Confocal (0.5AU) ○ ○ ○ ○ X
0.57 0.41 0.28 0.21 0.15 (μm3)
Large to small
d
SIM
Confocal (0.5AU)
YFP-H
Y
Z
X
e
SIM
Confocal (0.5AU)
YFP-H
1
2
3
4
f
1 2 3 4
SIM
Confocal (0.5AU)
0
-2.5
-5

Supplementary Figure 11

Computational geometry analysis of hippocampal neuron in brain slices

(a) An example of an in silico spine model viewed from different angles.

(b) Images of in silico spine models before and after convolution with Gaussian filters that mimic point spread function of SIM (FWHM; x-y 115 nm, z axis 270 nm) and confocal microscopy with a confocal aperture of 0.5 airy unit (AU) (FWHM; x-y 190 nm, z axis 410 nm).

(c) Surface mean curvature (pseudocolor mapping) of polygonal meshes generated from in silico model spines. Model spines with different sizes were generated by enlarging or shrinking the initial model proportionally. With smaller spines, nano-scale features were more difficult to detect after convolution with Gaussian filters. This analysis indicates that spines with their volumes larger than 0.18 μm^3 were suitable for detection of concave surfaces on spine heads.

(d) Representative x-y and x-z projection images of an identical apical dendrite with SIM and confocal microscopy of tissue slices containing YFP-expressing CA1 pyramidal neurons from adult *Thy1*-$H^{YFP/+}$ transgenic mice. Maximum intensity projection images were generated from the image stack with its total thickness of 7.56 μm. Bars: 2 μm in x-y and 1 μm in x-z.

(e) Higher-magnification views of the dendrite shown in panel (d). Bars: 2 μm in x-y.

(f) Surface mean curvature (pseudocolor mapping) of polygonal meshes created from dendritic spines labeled from 1 to 4 in panel (e). Negative curvature was detected on large spine heads (1 and 2) with both SIM and confocal microscopy.

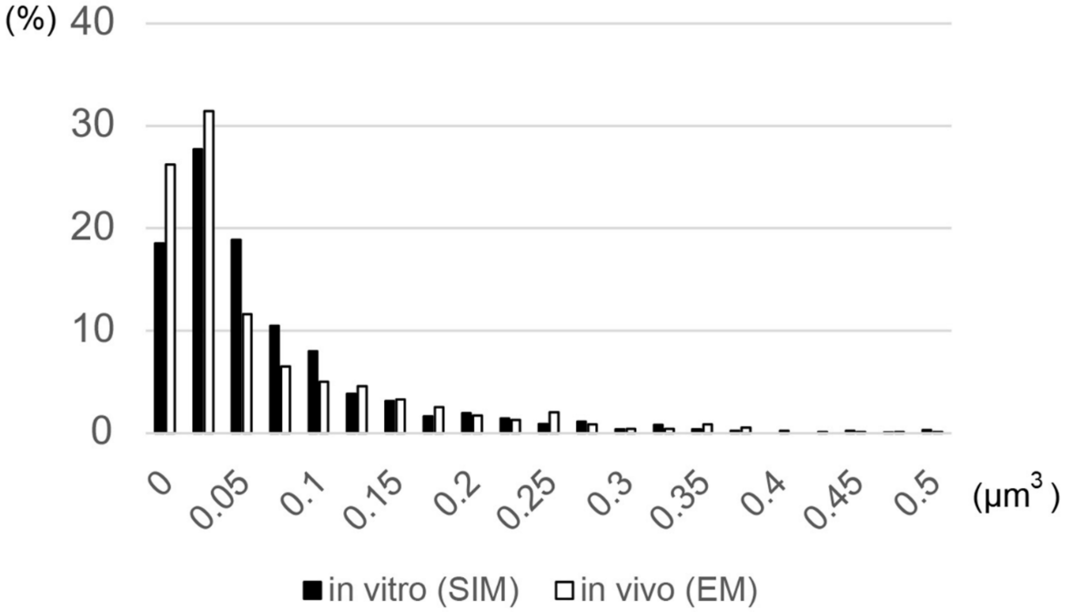

Supplementary Figure 12

Distribution of spine size estimated by SIM imaging of hippocampal neurons in culture and by EM reconstruction in vivo

Volume distribution of 1335 spines from hippocampal dissociated culture by SIM-based method and of 938 spines from hippocampal CA1 pyramidal neurons by EM-reconstruction method were compared. Tissues shrinkage is reported in the process of sample preparation for EM. A recent report directly compared chemical fixation and cryo- fixation and found about 26% volume shrinkage of the neuropil after chemical fixation. We set the conversion factor of 0.74 for tissue shrinkage in EM samples. We also set the conversion factor of 0.75 for overestimation of spine volume in SIM. This error is derived from lower resolution in the axial direction of SIM, which systematically extends objects in this direction. The conversion factor was determined from the comparison of SIM and EM data shown in Fig. 1e. After conversion of SIM data set using these two conversion factors, spine volume distributions were highly similar between hippocampal neurons in culture and CA1 pyramidal neurons in vivo. Spine volume of hippocampal neurons in culture measured by the SIM-based method was 0.079 ± 0.078 μm^3 (Mean ± SD, n = 1335). Spine volume measured by EM reconstruction of in vivo CA1 pyramidal cell dendrites was 0.076 ± 0.082 μm^3 (Mean ± SD, n = 938). By similar analysis of spine length, we found that spine length of cultured neurons measured with SIM was 1.01 ± 0.44 μm (Mean ± SD, n = 1335) and spine length of hippocampal CA1 neurons measured by EM reconstruction was 0.95 ± 0.42 μm (Mean ± SD, n = 100).

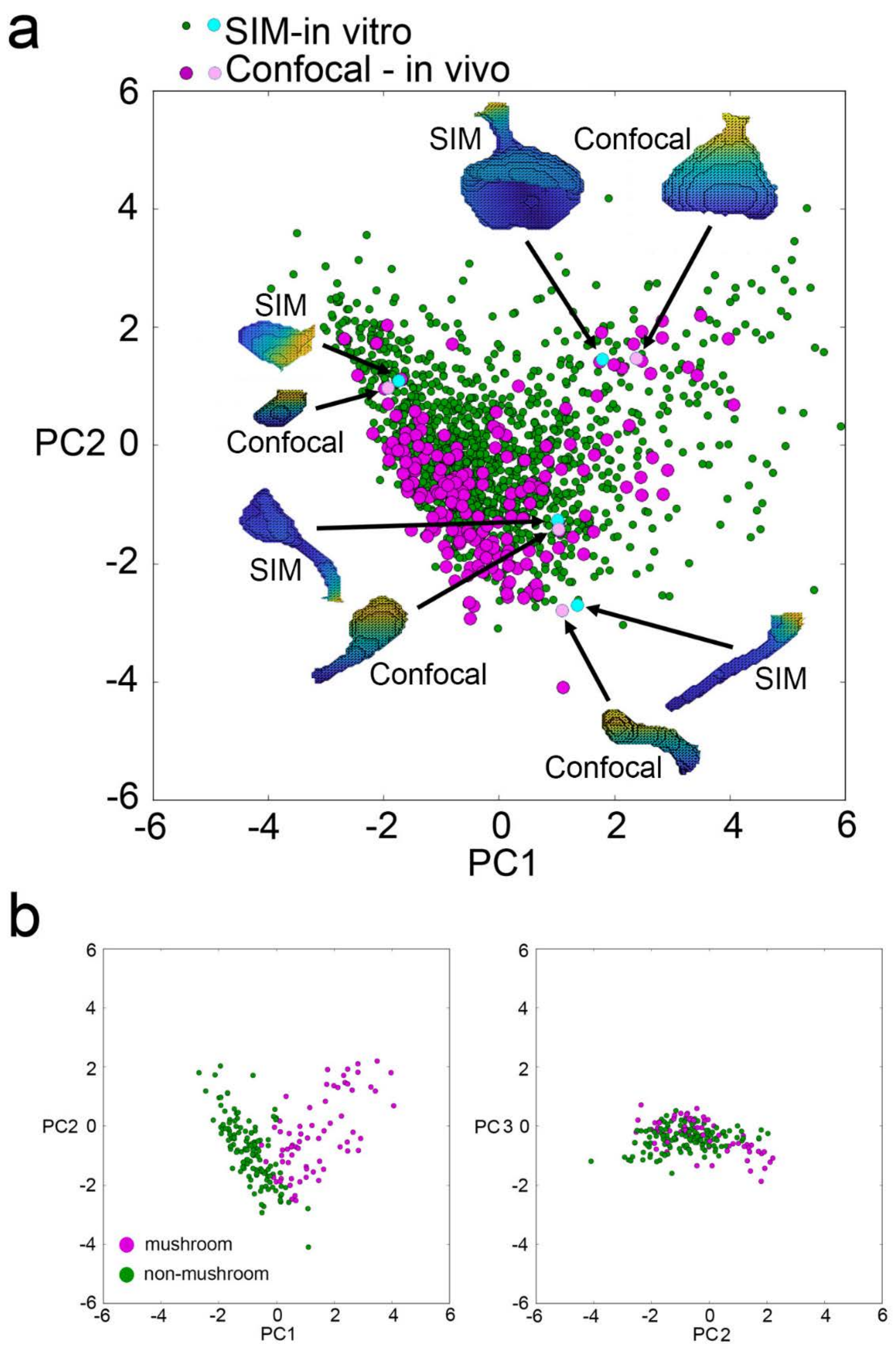
a
SIM-in vitro
Confocal - in vivo
SIM
Confocal
SIM
Confocal
SIM
Confocal
SIM
Confocal
PC2
PC1
b
mushroom
non-mushroom
PC2
PC1
PC3
PC2

Supplementary Figure 13

PCA and SVM-based analysis of in vivo spine population data

(a) PCA of spine shape data obtained by confocal microscopy of tissue slices expressing YFP in hippocampal pyramidal neurons. For PCA, the same projection matrix created for GFP-labeled culture neurons was applied to the spine data obtained from tissue slices. Distribution of spine data from cultured neurons imaged by SIM and from tissue slices imaged by confocal microscopy was similar and the positions of typical mushroom, thin, and small spines were preserved in the feature space (n = 165 for tissue slices, n = 1335 for cultured neurons).

(b) SVM-based classification of spine data from tissue slices imaged by confocal microscopy. The SVM classifier trained with the data set obtained from GFP-expressing cultured neurons was applied to the data set from the tissue slices (n = 165). Classification accuracy was 88.0%, indicating slight decline in performance in comparison with the data obtained from cultured neurons (89.3%).

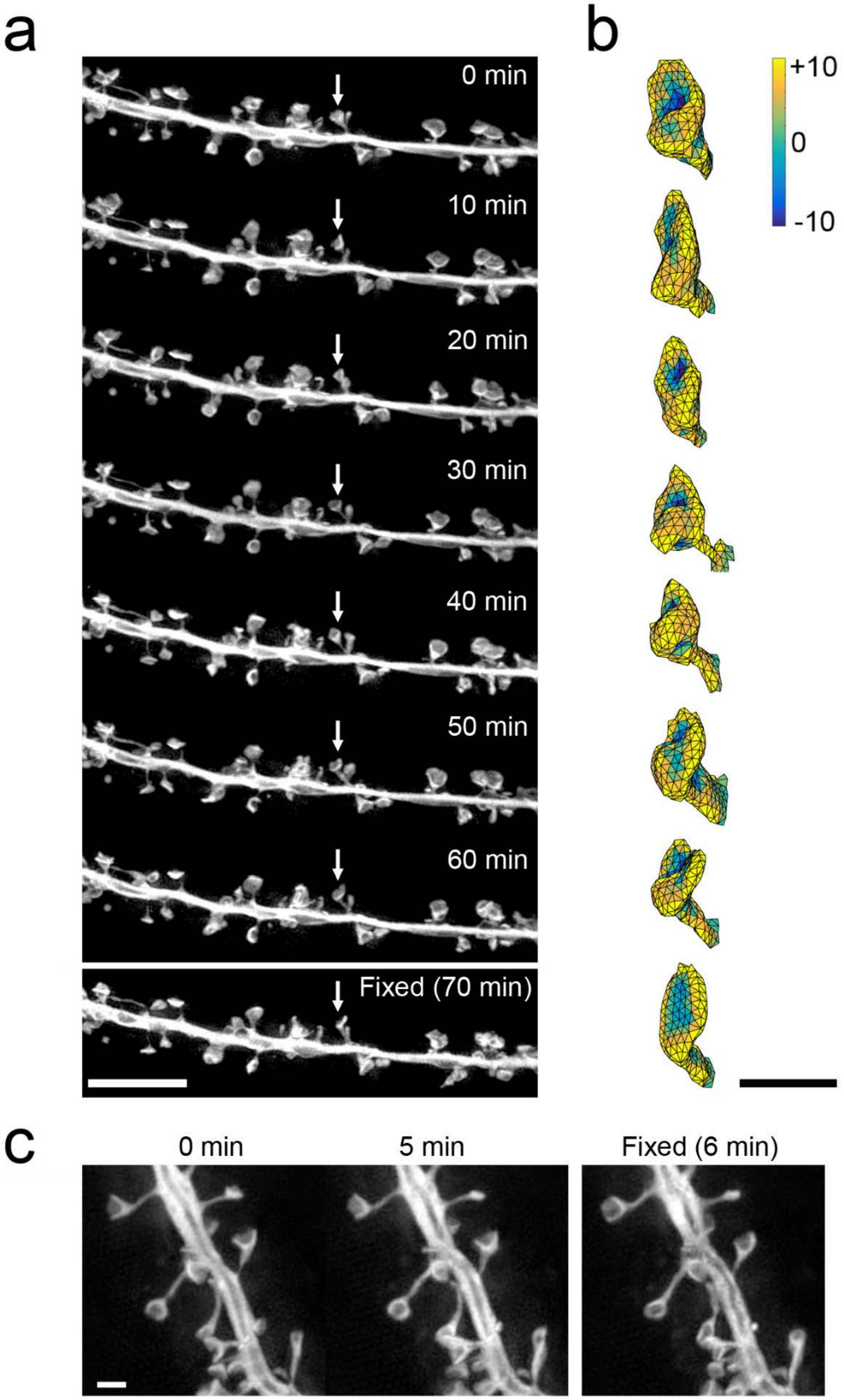
a
0 min
10 min
20 min
30 min
40 min
50 min
60 min
Fixed (70 min)
b
+10
0
-10
c
0 min
5 min
Fixed (6 min)

Supplementary Figure 14

Time-lapse data acquisition of 3D-SIM

(a) Original 3D-SIM images taken at intervals of 10 min for a total period of 60 min. After image acquisition, the sample was fixed at t = 70 min, and the 3D-SIM image was again obtained. Bar: 4 μm.

(b) Reconstructed polygonal meshes of a single spine (arrows in panel (a)). Dynamic changes in spine head morphology were detected. Bar: 1 μm.

(c) Live 3D-SIM images taken at 0 min and 5 min. The sample was fixed at t = 6 min, and the 3D-SIM image was again obtained. Bar: 1 μm.

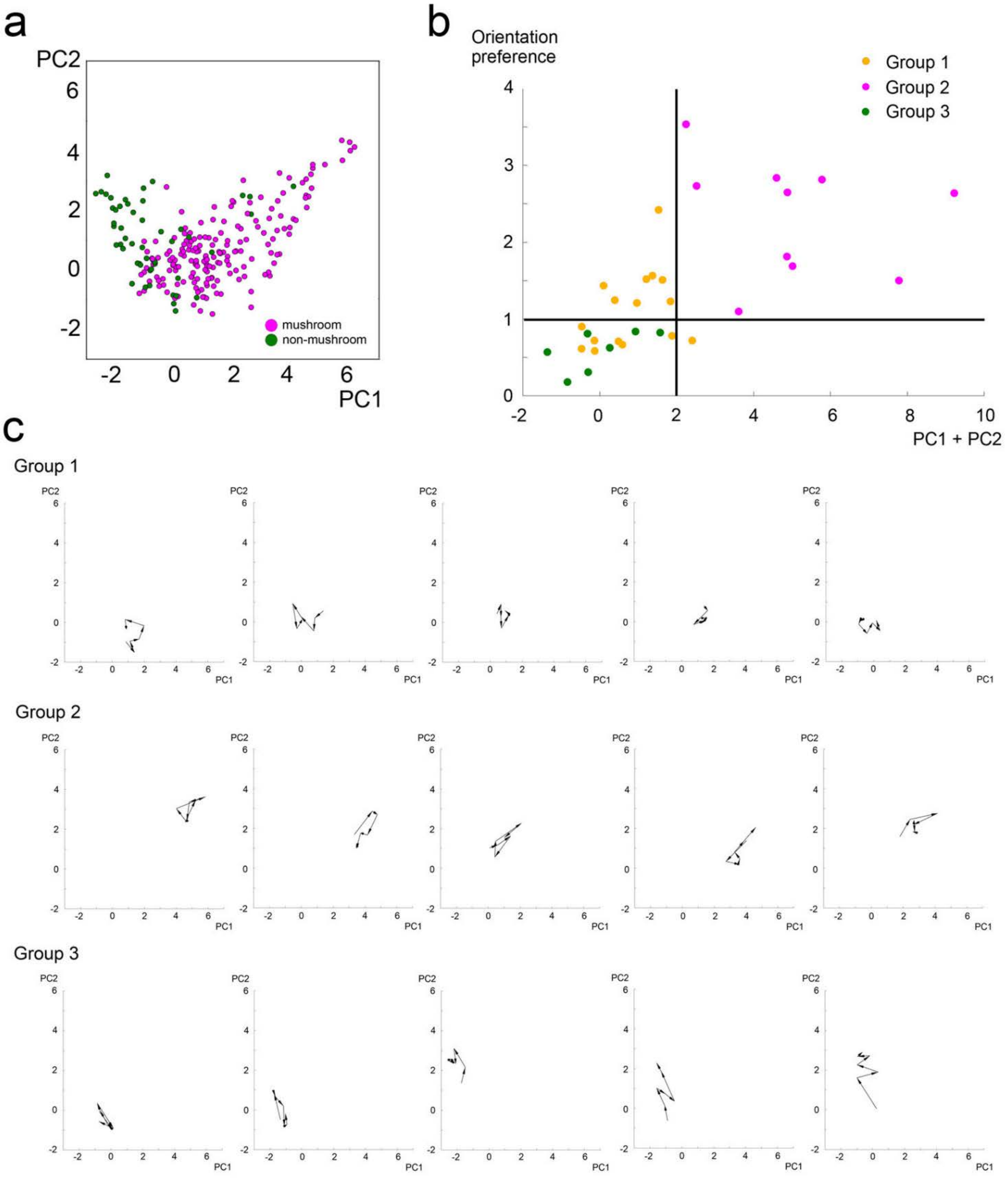
a
PC2
PC1
mushroom
non-mushroom
b
Orientation preference
Group 1
Group 2
Group 3
PC1 + PC2
c
Group 1
Group 2
Group 3

Supplementary Figure 15

Classification of group 1, 2, and 3 spines

(a) SVM-based classification of 33 spines imaged at 10 min intervals for 60 min. If an identical spine was classified as mushroom or non-mushroom at different time points, the class with a larger number of time frames was selected as the representative class in the subsequent analysis.

(b) Classification of spines by their positions in the feature space and the orientation of spine shape trajectories. The sum of PC1 and PC2 values reflects spine size and roundness. Orientation preference reflects the extent of shape transition along the axis 45 degrees counterclockwise from the PC1 axis. This orientation is along the axis from small/thin to large/round spines. Group 2 spines (classified as mushroom spines by SVM, with PC1+PC2>2 and Orientation preference>1) are large and round mushroom spines, and their shapes change from small/thin to large/round. Group 1 spines (classified as mushroom spines by SVM, with PC1+PC2<2 or Orientation preference<1) are also mushroom spines, but they are smaller and thinner, and their shape transition is less oriented. Group 3 spines are spines classified as non-mushroom by SVM.

(c) Examples of spine shape changes in the plane of PC1 and PC2 for three spine classes. Three groups show distinct trajectories of shape transition.
