# Supplementary material for: Computational geometry analysis of dendritic spines by structured illumination microscopy
Source: Nat Commun. 2019 Mar 20;10:1285. doi: 10.1038/s41467-019-09337-0 (PMC6427002; doi:10.1038/s41467-019-09337-0)
Supplement: Supplementary file 3 — Reporting Summary [file 41467_2019_9337_MOESM3_ESM.pdf]

nature research

Corresponding author(s): Shigeo Okabe

Last updated by author(s): Feb 5, 2019

Reporting Summary

Nature Research wishes to improve the reproducibility of the work that we publish. This form provides structure for consistency and transparency in reporting. For further information on Nature Research policies, see Authors & Referees and the Editorial Policy Checklist.

Statistics

For all statistical analyses, confirm that the following items are present in the figure legend, table legend, main text, or Methods section.

n/a	Confirmed	
☐	☒	The exact sample size (*n*) for each experimental group/condition, given as a discrete number and unit of measurement
☐	☒	A statement on whether measurements were taken from distinct samples or whether the same sample was measured repeatedly
☐	☒	The statistical test(s) used AND whether they are one- or two-sided<br>*Only common tests should be described solely by name; describe more complex techniques in the Methods section.*
☒	☐	A description of all covariates tested
☒	☐	A description of any assumptions or corrections, such as tests of normality and adjustment for multiple comparisons
☐	☒	A full description of the statistical parameters including central tendency (e.g. means) or other basic estimates (e.g. regression coefficient) AND variation (e.g. standard deviation) or associated estimates of uncertainty (e.g. confidence intervals)
☐	☒	For null hypothesis testing, the test statistic (e.g. *F*, *t*, *r*) with confidence intervals, effect sizes, degrees of freedom and *P* value noted<br>*Give P values as exact values whenever suitable.*
☒	☐	For Bayesian analysis, information on the choice of priors and Markov chain Monte Carlo settings
☒	☐	For hierarchical and complex designs, identification of the appropriate level for tests and full reporting of outcomes
☒	☐	Estimates of effect sizes (e.g. Cohen's *d*, Pearson's *r*), indicating how they were calculated

Our web collection on statistics for biologists contains articles on many of the points above.

Software and code

Policy information about availability of computer code

Data collection	Stack algorithm V2.10 of NIS-Elements AR (NIKON) was used for SIM image reconstruction.
Data analysis	Data analysis was performed with Python and MATLAB R2017b. Following MATLAB functions were used for analysis; plyread version 1.0 by yesmine and MarchingCubes version 1.3 by Peter Hammer. Custom scripts written in MATLAB are available as supplementary programs.

For manuscripts utilizing custom algorithms or software that are central to the research but not yet described in published literature, software must be made available to editors/reviewers. We strongly encourage code deposition in a community repository (e.g. GitHub). See the Nature Research guidelines for submitting code & software for further information.

Data

Policy information about availability of data

All manuscripts must include a data availability statement. This statement should provide the following information, where applicable:

- Accession codes, unique identifiers, or web links for publicly available datasets
- A list of figures that have associated raw data
- A description of any restrictions on data availability

All data files are available from the corresponding author on reasonable request.

Field-specific reporting

Please select the one below that is the best fit for your research. If you are not sure, read the appropriate sections before making your selection.

☒ Life sciences ☐ Behavioural & social sciences ☐ Ecological, evolutionary & environmental sciences

For a reference copy of the document with all sections, see nature.com/documents/nr-reporting-summary-flat.pdf

Life sciences study design

All studies must disclose on these points even when the disclosure is negative.

Sample size	No statistical methods were used to predetermine sample-size, but our sample sizes are similar range to those reported in previous publications (ref 7, 23)
Data exclusions	In some cases, polygon meshes of a pair of nearby spines were merged; these were omitted from the analyses.
Replication	Attempts at replication were successful. The number of replicates for each experiment is presented in the figure legends.
Randomization	For neuronal culture from genetically modified mouse experiment, cells were randomly chosen and analyzed.
Blinding	Data collection and were not performed blind to the conditions of the experiments. Data analysis was performed in objective and standardized way.

Reporting for specific materials, systems and methods

We require information from authors about some types of materials, experimental systems and methods used in many studies. Here, indicate whether each material, system or method listed is relevant to your study. If you are not sure if a list item applies to your research, read the appropriate section before selecting a response.

Materials & experimental systems

n/a	Involved in the study	
☒	☐	Antibodies
☒	☐	Eukaryotic cell lines
☒	☐	Palaeontology
☐	☒	Animals and other organisms
☒	☐	Human research participants
☒	☐	Clinical data

Methods

n/a	Involved in the study	
☒	☐	ChIP-seq
☒	☐	Flow cytometry
☒	☐	MRI-based neuroimaging

Animals and other organisms

Policy information about studies involving animals; ARRIVE guidelines recommended for reporting animal research

Laboratory animals	ICR mice (Japan SLC), heterozygous synGAP mutant mice , homozygous knock-in mice harboring a knock-in of a kinase-dead K42R mutation in Ca2+/calmodulin-dependent protein kinase IIα were used for neuronal culture at embryonic day 16.5.<br><br>Male transgenic mice (Thy1-H YFP/+, 3 months old) was used for tissue preparation.
Wild animals	No wild animals were used.
Field-collected samples	No field-collected samples were used.
Ethics oversight	All animal experiments were approved by the animal welfare ethics committee of the University of Tokyo.

Note that full information on the approval of the study protocol must also be provided in the manuscript.
