# Supplementary material for: Computational geometry analysis of dendritic spines by structured illumination microscopy
Source: Nat Commun. 2019 Mar 20;10:1285. doi: 10.1038/s41467-019-09337-0 (PMC6427002; doi:10.1038/s41467-019-09337-0)
Supplement: Supplementary file 4 — Description of Additional Supplementary Information [file 41467_2019_9337_MOESM4_ESM.pdf]

Description of Additional Supplementary Files

File Name: Supplementary Software 1
Description: Supplementary programs contain 4 custom scripts for MATLAB and 5 custom MATLAB functions necessary for running scripts. In addition, 2 open source MATLAB functions, “plyread” and “MarchingCubes” are required. The details of these codes are described in the file “About supplementary programs”.
