# Supplementary material for: Computational geometry analysis of dendritic spines by structured illumination microscopy
Source: Nat Commun. 2019 Mar 20;10:1285. doi: 10.1038/s41467-019-09337-0 (PMC6427002; doi:10.1038/s41467-019-09337-0)
Supplement: Supplementary file 5 — Supplementary Software 1 [file 41467_2019_9337_MOESM5_ESM.lzh]

Supplementary programs

1. **SIM_activecontour**: the custom script for processing SIM image stacks and generation of binary image stacks.
2. **SIM_spine_detection**: the custom script for spine detection and polygon mesh generation.
3. **Geometric_calculation_program**: the custom script for calculation of geometrical parameters, including spine length, spine surface area, spine volume, and other more complex morphological features.
4. **Geometric_curvature_program**: the custom script for calculation of mean and Gaussian curvature.
5. **RotateShaftHorisontal**: the custom MATLAB function for adjustment of dendritic shaft orientation. (necessary for running SIM_spine_detection)
6. **FitMainDendrite**: the custom MATLAB function for rough estimation of dendritic shaft volume. (necessary for running SIM_spine_detection)
7. **RefineSpineLocation**: the custom MATLAB function for refinement of dendritic shaft volume and determination of voxels of spines. (necessary for running SIM_spine_detection)
8. **SpineMeshCalc**: the custom MATLAB function for generation of polygon mesh data of spines. (necessary for running SIM_spine_detection)
9. **DeleteIsolateSurface**: the custom MATLAB function for deletion of meshes isolated from main mesh structures. (necessary for running SIM_spine_detection)

In addition, following MATLAB functions are required for running the above programs.

10. **plyread**: necessary for reading PLY files. Version 1.0 by yesmine.
    https://jp.mathworks.com/matlabcentral/fileexchange/47484-plyread-m

11. **MarchingCubes**: necessary for generating polygon mesh data. Version 1.3 by Peter Hammer.
    https://jp.mathworks.com/matlabcentral/fileexchange/32506-marching-cubes
